# Congenital Absence of Pericardium: The Swinging Heart

**DOI:** 10.3390/jimaging10080199

**Published:** 2024-08-14

**Authors:** Raffaella Marzullo, Alessandro Capestro, Renato Cosimo, Marco Fogante, Alessandro Aprile, Liliana Balardi, Mario Giordano, Gianpiero Gaio, Gabriella Gauderi, Maria Giovanna Russo, Nicolò Schicchi

**Affiliations:** 1 Pediatric Cardiology, University of Campania ‘Luigi Vanvitelli’, Former Second University of Naples, Monaldi Hospital-AORN Ospedali dei Colli, 81100 Naples, Italy; ra.marzullo@gmail.com (R.M.); renato.cosimo21@gmail.com (R.C.); giordanomario1123@gmail.com (M.G.); gianpierogaio@hotmail.com (G.G.); gabriella.gauderi@libero.it (G.G.); mgiovannarusso@gmail.com (M.G.R.); 2 Department of Pediatric and Congenital Cardiac Surgery and Cardiology, University Hospital of Marche, 60126 Ancona, Italy; alessandro.capestro@ospedaliriuniti.marche.it; 3 Department of Radiology, Maternal-Child, Senological, Cardiological Radiology and Outpatient Ultrasound, University Hospital of Marche, 60126 Ancona, Italy; 4 Department of Cardiology, Ospedale del Mare, 80147 Naples, Italy; ale_aprile@yahoo.it; 5 Health Professions Area and Diagnostic Technical Area, University Hospital of Marche, 60126 Ancona, Italy; liliana.balardi@ospedaliriuniti.marche.it; 6 Cardiovascular Radiological Diagnostics, Department of Radiology, University Hospital of Marche, 60126 Ancona, Italy; nicolo.schicchi@ospedaliriuniti.marche.it

**Keywords:** congenital pericardium absence, congenital disease, echocardiography, cardiac computed tomography, cardiac magnetic resonance

## Abstract

Congenital absence of the pericardium (CAP) is an unusual condition discovered, in most cases, incidentally but can potentially lead to fatal complications, including severe arrhythmias and sudden death. Recently, the use of modern imaging technologies has increased the diagnosis of CAP, providing important findings for risk stratification. Nevertheless, there is not yet consensus regarding therapeutic decisions, and the management of patients with CAP remains challenging. In this paper, we discuss the pathophysiological implication of CAP, review the current literature and explain the role of multimodality imaging tools for its diagnosis, management and treatment.

## 1. Introduction

Congenital absence of the pericardium (CAP) is a rare cardiac malformation frequently associated with other thoraco-abdominal defects and congenital heart diseases. Both location and size of the defect affect variably thoracic organ interactions and could result in geometric distortion of ventricles, great vessels or coronary arteries. The broad spectrum of clinic presentations ranges from asymptomatic cases to fatal presentations that could mimic other cardiovascular diseases [1,2,3].

## 2. Embryology

Failed mesodermal development has been assumed as an embryologic mechanism for CAP [4,5]. The magnitude and affected side of aberrant mesodermal development cause the different anatomic variants of CAP ranging from partial forms to complete defects with unilateral or bilateral distribution [6]. In most cases, the premature atrophy of the duct of Cuvier is the “primum movens” for failure of pericardial closure. It occurs most commonly on the left side, justifying the large predominance of the left defect [5,6,7]. In the extreme form, the anomalous compartmentation results in the persistence of peritoneal–pericardial communication, which could hesitate in the eventration of the diaphragm and the heart outside the thoracoabdominal wall (ectopia cordis) alone or with other viscera inside [1]. Even more rarely, the defects of the diaphragmatic pericardium can lead to the herniation of the greater omentum inside the pericardial cavity [8].

## 3. Incidence

Current data on CAP are derived from isolated case reports and small case series. Analysis of published data reveals that the diagnosis has been made across different age groups, from fetus to octogenarian. The incidence is estimated between 0.007% and 0.044% [6,9,10], but the real burden of diseases is probably higher given that most patients are asymptomatic and that CAP is usually an incidental finding in imaging studies.

A male predominance has been reported for CAP [2]. Complete left-sided defects are the commonest reported anatomic variants, representing 70% of all pericardial defects. Right-sided defects account for 17% of all defects, while the complete bilateral absence of pericardium represents 9% of the disease. Other defects are exceedingly rare [2,11].

Although CAP is usually an isolated defect, one-third of patients have associated congenital anomalies, including atrial septal defects, patent ductus arteriosus, atrioventricular valve diseases, tetralogy of Fallot and partial anomalous pulmonary venous drainage [2]. Extra-cardiac malformations have also been described with CAP, such as diaphragmatic hernia, lung hypoplasia and bronchogenic cyst [10,12]. Additionally, CAP has been detected in patients with Marfan syndrome or other aortic connective tissue disorders [13,14].

To date, only one case of the three-generation familial presentation of partial absence of the left pericardium has been reported in the literature [15].

## 4. Pathophysiologic Implications

The pericardium plays many physiologic roles in cardiac mechanics, supporting the heart’s position in the thorax and providing a physical barrier to the cardiac surface.

With the pericardium, the heart makes a rotation along its long axis during the systole that culminates in the twisting motion of the left ventricle (LV). This corresponds to a clockwise rotation of the base and counter-clockwise torsion of the apex. In the diastole, the potential energy stored during the twisting is released, leading to a reverse rotation (untwist) that contributes to the diastolic filling [16].

In the absence of pericardium, the anchoring and cushioning function decreased, affecting the functional rotation of LV. In fact, the LV shows only the apical counterclockwise rotation without effective clockwise rotation of the cardiac base. This results in the exaggerated anterior swinging motion of LV during the systole and, consequently, an extreme dorsal displacement of the cardiac apex during the diastole to restore its normal position. These dynamic movements are markedly evident in the left lateral decubitus position due to loss of pericardial support and less pronounced in the right lateral decubitus position because lying on the right side makes the heart itself move anteriorly in the thorax and limits the anterior motion of the heart in systole [16].

Postural changes from the left to right lateral decubitus also have prominent implications on right ventricle (RV) filling and, consequentially, on ventricular coupling in patients with pericardial defects. Lacking pericardial restraint, the overstated elevation of hydrostatic forces and reduced excursion of the tricuspid ring affect the systemic venous return, mimicking an acute volume overload of the right ventricular cavity when the patients lay on their left. In this position, increased RV volume shifts leftward the interventricular septum, impairing the LV diastolic pression–volume curve as seen in decreasing diastolic filling and its developed pressure [17]. In this context, the distortion of ventricular geometry can lead to progressive traction of the chordal apparatus of atrioventricular valves that might contribute to both tricuspid and mitral regurgitation [6].

Finally, abnormal twisting of the heart and strain on the great vessels and coronary arteries might also exist in ischemia and even in herniation and the incarceration of cardiovascular structures in the case of a partial defect [18,19].

## 5. Clinical Presentation

The broad spectrum of the clinic presentation of CAP ranges from asymptomatic cases to fatal complications. In most cases, CAP has a reduced clinical relevance, and it is detected as an incidental finding in imaging studies or during surgery or necropsy [6,7,20,21].

Patients with partial defects are more symptomatic and at higher risk for complications than those with complete bilateral or complete left-sided absence of the pericardium [2]. Paroxysmal atypical chest pain, often nocturnal or postural, with variable intensity, is the commonest presentation [5]. This may be associated with the compression of coronaries by the edge of the residual pericardium. Otherwise, atherosclerotic coronary artery disease can coexist with CAP [7].

Dyspnea and trepopnea are also reported in patients with CAP, referring to several mechanisms, such as compression of the left lower pulmonary vein between the left atrium and descending aorta or spine and the acute volume loading of the ventricles and hypoplasia of the left lung. Patients with localized forms also reference palpitations and syncope [5].

More rarely, dramatic presentation has been described in patients with CAP. Strangulation of the left ventricle across the defect, incarceration of the myocardium (commonly of the left atrial appendage), torsion of great vessels or coronary compression have been reported in patients experiencing sudden death [18,22,23,24]. Finally, aortic dissection due to torsion of the great vessel has also been documented [14].

Physical examination in patients with CAP is not characteristic. Sometimes, apical impulses can be palpated in the left axilla, and a systolic ejection murmur can be detected at the cardiac base.

## 6. Electrocardiography

Typically, the electrocardiography (EKG) reveals an incomplete right bundle branch block associated with right QRS axis deviation and a leftward displacement of the precordial transitional zone [2]. Additional findings include electrical alternans due to the beat-to-beat swinging motion of the heart and ST elevation in patients experiencing acute chest pain [7,23]. Finally, paroxysmal atrial fibrillation has been described in a patient with herniation of the left atrial appendage [25], and ventricular fibrillation has also been reported in patients with myocardial ischemia or strangulation of LV [26].

## 7. Non-Invasive Imaging

### 7.1. Chest X-ray

**Figure 1:**
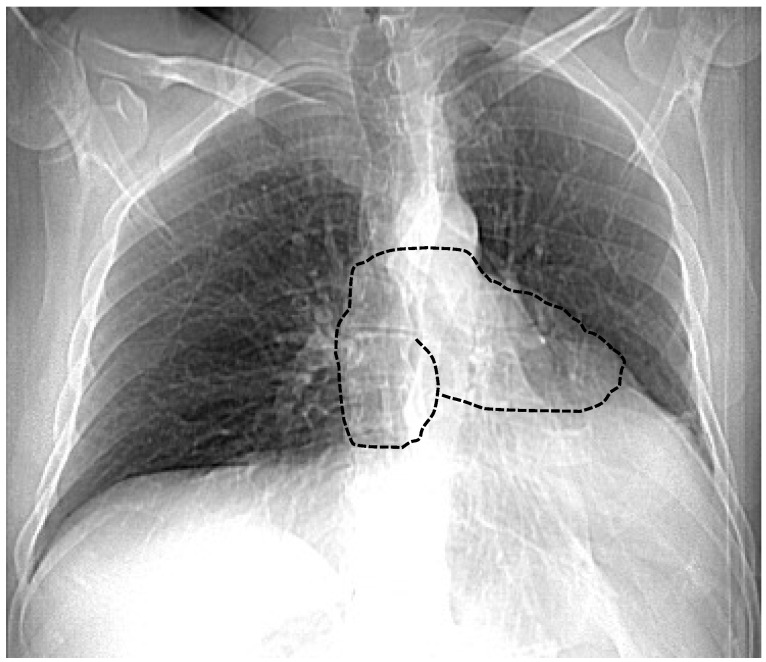
Chest X-ray of a patient with CAP. The right heart border is obscured by vertebral spine while the left edge results in fattening and elongation; this is called the “Snoopy signs” (drawn in black line).

Chest X-ray is an important tool in support of a diagnosis of CAP. In a large pericardial defect, the cardiac silhouette is markedly levopositioned and shows a posterior displacement into the mediastinum (without tracheal compression) in the postero-anterior projection. In this condition, the right heart border is obscured by the vertebral spine, while the left edge results in fattening and elongation; this is called “Snoopy signs” (Figure 1). A prominent radiolucent area referring to lung tissue interposition between the ascending aorta and the pulmonary trunk is noted in the case of the absence of a homolateral pleura. Although a chest X-ray is usually unremarkable in small pericardial defects, the bulging of the left upper heart border related to a herniated left atrial appendage can be shown in the presence of a foramen-type defect [2].

### 7.2. Echocardiography

Echocardiography provides indirect but not specific clues for CAP. Failure to obtain a standard view by usual acoustic windows is a distinct echocardiographic feature of complete CAP. Lateral placement of the transducer may be necessary to obtain both parasternal and apical views. In some cases, echocardiographic interrogation may be possible only in supine or right-side lateral decubitus. In the left parasternal window, levoposition results in an unusual oblique and posterior orientation of the cardiac apex. The paradoxical wall motion of interventricular septum and RV dilatation may mimic right volume overload or the “septal bounce” and RV predominance detected in individuals with pectus excavatum [27]. Exaggerated diastolic flattening of posterior LV may also be noted and may be explained with deeper translocation of the heart into the thorax. Interestingly, these findings may disappear with the change of position according to physiopathological considerations dissuading to other diagnoses. In addition, the epicardial–pericardial interface cannot be well visualized in two-dimensional or M-mode imaging. In the apical window, the heart acquires a “teardrop” configuration characterized by bulbous LV and elongated atria. Cardiac hypermobility with swinging motion is evident [2,3]. Both tricuspid and mitral regurgitation due to functional annular distortion can be seen in patients with CAP [6]. Pulse Doppler pulmonary veins may demonstrate a reduced systolic-to-diastolic ratio, consistent with a decrease in pericardial pressure during ventricular ejection. Similarly, the reduction of the systolic flow in the superior vena cava can be detected [2,3].

LV torsion by 2D-speckle tracking echocardiography appears decreased in contrast to normal longitudinal, radial and circumferential systolic strains. The pendulum movement of the heart and electrical alternans may also be documented during stress echocardiography [28].

Stress echocardiography demonstrated mild RV dilatation at rest and an increase in RV volume directly after exercise [29,30].

### 7.3. Cardiac Magnetic Resonance Imaging

Cardiac Magnetic Resonance imaging (CMR) is the best method for the diagnosis of CAP. Due to its high contrast and temporal resolution, it allows soft tissue definition and better anatomic viewing. The CMR examination must include T1-weighted (T1W) and cine sequences for direct pericardial imaging and functional assessment. Images should be obtained in multiple planes to maximize diagnostic accuracy. A complete exam would include whole heart coverage in the axial and sagittal or coronal planes and selected vertical long-axis and short-axis views. Real-time images should be obtained in the four-chamber and short-axis orientation to assess for paradoxical septal motion [31].

**Figure 2:**
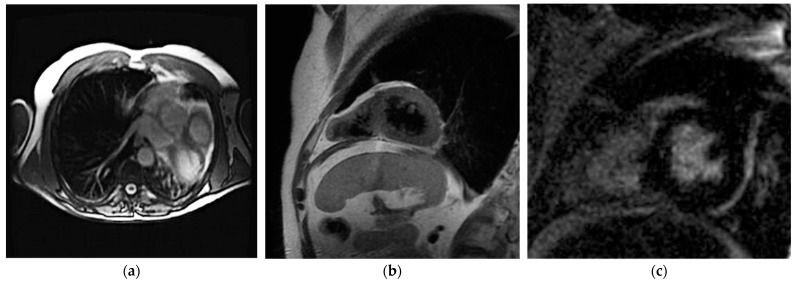
CMR of a patient with CAP. The axial view demonstrates the heart’s displacement to the left hemithorax (**a**); in T1W and late gadolinium enhancement, short-axis view (**b**,**c**) confirmed the absence of pericardium.

The heart’s displacement to the left hemithorax and lung tissue interposition among heart and vessels are suggestive findings of pericardium absence. CMR cine-sequences can reveal functional abnormalities of the heart, like the excessive systo-diastolic excursion of the apex. Disproportionated systo-diastolic changes can also be quantified by parameters like whole-heart volume change (WHVC), expressed as the systo-diastolic variation of heart volume measured in axial cine-sequences. A WHVC > 13% threshold had a diagnostic accuracy with sensitivity and specificity of 100%. T1W sequences finally confirm the lack of pericardium and allow the distinction between partial and complete CAP [31]. Visualization is best during systole when epicardial and pericardial fat layers are present [32]. Figure 2 shows CMR images of a patient with CAP.

Finally, CMR using additional methods, such as 4D-flow, can detect associated congenital valvular anomalies by evaluating alterations of cardiac flow [33]. Although the use of a contrast medium is not necessary for the identification of CAP, the differential diagnosis is usually broad, and it may still be preferable to use a contrast protocol to maximize the information obtained, such as the detection of pericardial inflammation if it exists a clinical suspicion of pericarditis [34].

### 7.4. Cardiac Computed Tomography

Cardiac computed tomography (CCT) has shown an important diagnostic role in pericardium defects for the higher sensitivity to identify indirect signs, such as the excessive leftward rotation of the heart, the interposition of lung tissue in the areas of absent pericardium as well as the bulging of the left atrial appendage through the defect [34].

Given its high spatial resolution, CCT is more sensitive than MRI to detect morphological abnormalities of the pericardium, especially in the case of small and incomplete defects, and allows for ruling out any herniation of the heart chambers or coronary and large mediastinal vessels. The use of intravenous contrast allows for detailed evaluation of coronary anatomy and heart chamber size.

CCT offers speed and excellent spatial resolution but involves radiation exposure compared to MRI [34]. However, with recent technological improvements, radiation exposure during CCT scans has been significantly minimized [35]. These improvements include the use of an ultra-high pitch protocol, the use of low kilovoltages, especially in pediatric patients and the application of new iterative image reconstruction algorithms for better image quality at the same radiation dose [35,36].

The direct visualization of the pericardium can be challenging in patients with minimal adjacent fat, such as children or young adults [37]. Moreover, CCT can detect high-risk features such as coronary artery compression, atrial appendage protrusion and myocardial deformation. However, the heart’s swinging motion due to CAP can create artifacts in CCT images, leading to potential misdiagnoses. To be able to identify and measure the heart’s movement during the systole and diastole, a cardiac CT acquisition with ECG synchronization is recommended [38].

**Figure 3:**
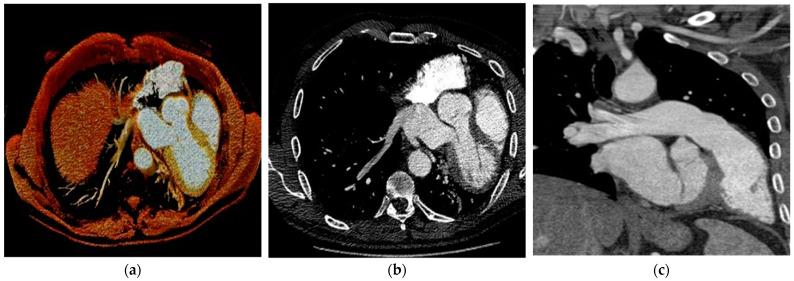
CCT of a patient with CAP. Panels (**a**–**c**) demonstrate extreme cardiac levorotation in axial and coronal views.

Finally, the use of new techniques, such as dual-energy, can allow the evaluation of the mediastinum and the perfusion of the lung parenchyma through the creation of iodine maps. In addition, photon-counting technology significantly improves the spatial resolution of CT images and could allow the visualization of the pericardium or diagnose its absence [31,36,39]. Figure 3 shows CCT images of a patient with CAP. Table 1 summarizes the different modalities for CAP diagnosis.

## 8. Diagnostic Approach and Management

Due to the lack of standardized diagnostic approaches and best treatment practices, personalized case-by-case strategies are used. Usually, the management of CAP is based on clinical presentation and the size of the defect.

The complete absence of pericardium is usually asymptomatic. The literature reports only one case of sudden cardiac arrest in complete absence of pericardium [26]. Notably, in this case, no signs of acute or chronic ventricular herniation have been detected. Studies of cardiac function showed that left ventricular ejection fraction in patients with complete CAP does not differ from healthy controls as well as life expectancy is the same as those with a normal pericardium [10,40]. However, an increased risk for traumatic type A aortic dissection has been reported [13,14]. These arguments support conservative management for complete forms of CAP, limiting the surgical approach in the case of refractory symptoms and/or complications [41].

Conversely, management and therapeutic strategies for small- and moderate-sized partial defects are still debated. Despite most of the evidence supporting a conservative approach, some authors suggest prophylactic surgery regardless of the clinical presentation to prevent the risk of herniation and strangulation. Recently, the detection of high-risk anatomic features by multimodality cardiovascular imaging, including (1) LV myocardial crease or hinge point on echocardiography, CMR or CCT; (2) coronary compression on CCT; (3) inducible ischemia on stress perfusion imaging (SPECT, PET or CMR) and (4) evidence of left atrial appendage herniation have been proposed to risk stratification. Detection of these “red flags” may be used to tailor surgical planning [27].

Pericardiotomy is the most used surgical technique with good clinical outcomes. Other techniques include pericardioplasty (patch closure or enlargement of the defect excision of the herniated atrial appendage) and division of adhesions [4].

To date, there are no specific recommendations regarding physical activity in patients with CAP. Extreme and contact sports should probably be discouraged, although recreational activities might be allowed in these patients [27].

## 9. Conclusions

CAP is a rare condition, usually undetected due to lack of symptoms, but that may become manifest with life-threatening complications, including sudden cardiac death. It may also mimic other cardiac diseases, leading to unnecessary diagnostic testing and potentially erroneous treatments. Echocardiography could support the diagnosis and suggest CCT and CMR scans to provide a definitive diagnosis and to identify the size and localization of the pericardial defect. Treatment strategies should be tailored case by case reserving the surgical option for symptomatic patients or those with high-risk features.

## Figures and Tables

**Table 1:** Diagnostic modalities for CAP detection.

Modality	Main Characteristics	Strengths	Limitations
Electrocardiography	Incomplete right bundle branch block Right QRS axis deviation Leftward displacement of the precordial transitional zone Peaked P waves Postural changes in the QRS vector	Availability Low cost Bed-side evaluation	No imaging evaluation
Echocardiography	Heart displacement Clockwise rotation Apparent right ventricular enlargement Paradoxical septal motion High movement of the heart	Availability Low cost High temporal resolution Bed-side evaluation	Operator dependent Low spatial resolution Low tissue characterization
Chest X-ray	Leftward heart displacement (Snoopy sign) Prominent main pulmonary artery Radiolucency between the heart and diaphragm	Availability Low cost Bed-side evaluation	Low sensibility Low specificity
CCT	Evaluation of heart position Evaluation of associated cardiac disease	Spatial resolution Temporal resolution	Radiation dose Low tissue characterization Artifact from metallic implantable devices
CMR	Direct pericardium visualization Evaluation of heart position Evaluation of heart function	Temporal resolution Tissue characterization	Long acquisition time Claustrophobia Presence of devices (compatibility, artifacts) High cost

Abbreviations. CCT: cardiac computed tomography; CMR: cardiac magnetic resonance.

## Data Availability

Dataset available on request from the authors.
